# The Capability Approach and the WHO healthy ageing framework (for the UN Decade of Healthy Ageing)

**DOI:** 10.1093/ageing/afad126

**Published:** 2023-10-30

**Authors:** Sridhar Venkatapuram, Jotheeswaran Amuthavalli Thiyagarajan

**Affiliations:** King’s College London, Global Health Institute, 1st Floor, Franklin Wilkins Building, Stamford Street, London SE1 9NH, UK; Ageing and Health Unit, Department of Maternal, Newborn, Child, Adolescent Health and Ageing, World Health Organization, CH-1211 Geneva, Switzerland

**Keywords:** geriatrics, gerontology, rehabilitation, occupational science & health, built environments and city planning, older people

## Abstract

This commentary discusses the WHO definition of health ageing in terms of functional abilities, and the problem definition and evidence-based public health response framework outlined in the 2015 WHO Report on Ageing and Health. After identifying the neglect of older people in health policy at national and global levels, some data are presented on the majority of COVID-19 deaths being older people. The discussion then focuses on the underlying ethical and analytical framework of functional abilities provided by the Capability Approach. The approach is presented as distinguishing between achievement and capability, the ethical significance of recognising both, and its inclusion of surrounding social conditions from local to global in assessing wellbeing of older people’s functional abilities. Measurement of functional abilities, informed by the Capabilities Approach, is stated to be an enormous and crucial task in establishing a global baseline, and making progress in improving the health and wellbeing of older people.

## Key Points

- Measurement framework for UN Decade of Healthy Ageing
- Applying Capability Approach to older people
- Health equity for older people worldwide

##  

In 2015, the World Health Organization (WHO) released the first World Report on Ageing and Health [1]. The following year, the WHO team behind the report published an article in the Lancet discussing the policy framework presented in the Report [2]. The team argued that the main policy problem related to ageing is that increasing longevity worldwide is not being accompanied by good health. The policy response, they further argued, requires a coherent and focused public health response that spans multiple sectors and stakeholders. And the 2015 WHO Report was presented as being just such a required evidence-based public health framework for robust action. And importantly, the Report was built around a new conception of ‘healthy ageing’ centred ‘on the notion of functional ability’. However, despite being described as just a notion, the concept of ‘functional ability’ (FA) presented in the Report is quite concrete. Also, unlike mere notions, the FA concept is grounded in and seeks to draw on the intellectual resources of an influential social justice theory and analytical framework for evaluating quality of life or wellbeing called the Capability/Capabilities Approach (CA) [3]. Understanding more this theoretical underpinning of FAs and the WHO policy framework is important as the UN Decade of Healthy Ageing (2021–2030) gets underway [4]. First, because one central and crucial effort is to measure and establish the global baseline of health of older people. And second, because we must then make progress in protecting, recovering and expanding the functional abilities of older people worldwide. Unlike a notion, a robust ethical and analytical theory can provide clarity and guidance regarding the ends and means of the UN Decade of Healthy Ageing as well as justify the transformational multi-sectoral policy framework put forward in the 2015 Report and advanced in subsequent WHO and United Nations (UN) meetings. This commentary discusses the WHO definition of healthy ageing and its underlying ethical and analytical framework with a view to helping ground research and discussions on measurement.

For those working in geriatrics and gerontology as well as related professions of rehabilitation, occupational medicine and science, and others, the WHO FA framework as well as the underlying CA theory may be unfamiliar. Moreover, despite high-level efforts at the UN and WHO that have produced policy recommendations, a global strategy, as well as initiated the Healthy Ageing Decade, there is little evidence that the FA framework or the related 2015 WHO policy recommendations are being implemented in low and middle-income countries (LMICs). The efforts are also not being visibly supported by international organisations and health development assistance programmes, or being taken up in evolving global health policy agendas [5]. Furthermore, the minimal engagement with the WHO’s FA model and Report’s recommendations is in line with a much greater and general lack of any meaningful recognition of the need for or prioritising of policies and programmes directed at protecting and improving health and wellbeing of older people, especially outside of the few highest-income countries.

The continued neglect of older people in health policy and planning should be particularly troubling given the vast majority of the millions of deaths and morbidity due to COVID-19 over the past three and a half years has been among older people. The Economist magazine currently estimates that excess deaths since COVID-19 began could be close to 30.4 million (with 95% CI) but presents information on deaths by age groups for only a few countries [6]. A recent 2023 study using WHO data sources concludes that persons aged ≥60 years accounted for more than 80% of COVID-19 deaths [7]. Moreover, primary COVID-19 vaccination series coverage among older people is stated as ranging from 90% (high-income countries) to 33% (low-income countries). Outside of the few high-income countries, most countries are far from the 100% coverage among older adults that the WHO recommends. While some high-income countries have declared the pandemic over, it is not clear when the number of COVID-19 deaths, especially among older adults, will reach a non-crisis level. The normalisation of higher levels of mortality due to COVID-19 (‘living with covid’) across all countries clearly implies making increased deaths of older people socially acceptable. In light of the longstanding neglect of older people in social and health policy, and the more recent acute number of deaths during the pandemic, it may now be more understandable why the WHO sought to link a global healthy ageing policy framework to an ethical theory addressing human wellbeing, moral rights and social justice.

## The Capability Approach and healthy ageing

The WHO defines healthy ageing as, ‘…the process of developing and maintaining the functional ability that enables wellbeing in older age. Functional ability is about having the capabilities that enable all people to be and do what they have reason to value…Functional ability consists of the intrinsic capacity of the individual, relevant environmental characteristics and the interaction between them’ [1]. This FA framework builds on the concept of ‘capability’ in the CA and related work on health capability and health justice [8].

The core idea of the CA is that human wellbeing or quality of life should be conceptualised in terms of their capabilities—what is practically possible for individuals to be and do in their daily lives. Such capabilities are made up by the individual’s bodily functioning, knowledge, and skills combined with their external social and physical conditions. The recognition of both internal and external components is crucial. For example, an older woman may have the *physical capacity (intrinsic)* to walk to the nearest village. However, she would not have practical possibility—the *capability*—if women are restricted from moving out of the house where she lives, there is no walkable path or accessible and safe mode of transportation. Take a second example. Although we are all advocating for greater access to healthcare for older people with neglected health conditions, the aim is not just to control their disease irrespective of anything else in their lives. Containing disease within the body has to align with supporting what the person wants to be and do in their daily living. The focus on capabilities arose from critiques of alternative ethical and policy approaches which focus on how happy the individual is, on satisfying her preferences, on her ownership of commodities, on her bodily functioning, or on her scores on indices that only partially capture all the dimensions of a decent human life. In contrast, capabilities are argued to be the right target of healthcare interventions and health policies.

A second important aspect of the CA is the distinction between capabilities and ‘functionings’ or achievements. While capabilities are what is practically possible for an individual to be and do in their daily life, a functioning is the realised outcome of the practically possibility. For example, the capability to be adequately nourished would lead to the functioning or outcome of being well nourished. The distinction is important for both conceptual analysis and ethical reasons. If we find that two individuals are severely malnourished, a focus just on outcomes would lead us to conclude both individuals are in equally bad states. However, if one was fasting as part of spiritual practice while the other could not afford to buy enough food, the two individuals have very different states of wellbeing. The person intentionally fasting is better off than the other. Tracking capabilities and functioning/outcomes enables us to recognise such important differences between individuals and implementing efficient policies.

The distinction between capabilities and outcomes also has ethical significance. In liberal societies as well as those that respect basic human rights, governments and other social institutions ensure that individuals are able to plan, pursue and revise their chosen way of living; they do not enforce one particular way of living or one defined set of physical and mental outcomes. By focusing on capabilities, social action becomes geared towards providing supportive social conditions that make every individual capable of living a decent life, and yet also free to choose if and when to realise their particular combination of capabilities and outcomes. The approach is centred on the important value of human freedoms which is globally shared. And, indeed, the CA recognizes that over the life course, and in some situations, the target of social action will be to produce functionings rather than capabilities (e.g. infants and children).

A third aspect of a capability focus is the CA requires and demands a greater attention to the profound influence of social conditions, from the local to the global, that impact an individual’s abilities to pursue or have good quality of life. It is not mere coincidence that researchers of social determinants of health (e.g. illness, disease and death) and health equity advocates have been analysing social inequalities and the social gradient in health in terms of the relative inequalities in the freedoms or opportunities of individuals and groups to live healthy and full lifespans [9]. There is no debate on whether healthcare is a profoundly important aspect of supportive conditions for good health outcomes and capabilities, but healthcare is just one of a wide range of social conditions that can develop, protect, expand, recover, or indeed, constrain and sometimes, extinguish capabilities. This expansion of the scope of analysis beyond the individual body to include surrounding environments when assessing health and wellbeing has been a longstanding pillar of disability advocates and the social model of disability [10].

A fourth aspect of the CA is the moral or ethical dimension. The approach robustly engages with concepts such as wellbeing, freedom, equality, human rights, social equity and justice. By conceptualising human wellbeing in terms of capabilities, human capabilities become moral goods. They are things that we can agree to ensure for each other as members of a good society or global community. Or, we can think of every human being as having moral rights to a set of basic capabilities that constitute a decent human life. Most national constitutions and international human rights law reflect just such rights to basic capabilities [11]. Seen in these moral terms, and recognising the sheer scale of preventable deaths, disability, mental suffering and constrained lives of older people and others because of unsupportive social choices and policy neglect, the calls for ‘health justice’ should now make more sense. So many geriatricians, gerontologists, academic panels and others have been identifying the enormous social neglect and harms older people are facing, and sometimes reaching for the language of human rights [12]. The WHO’s FA framework and its underlying CA theory provides formidable intellectual resources to support such intuitions, analyses, and advocacy.

## Measurement and monitoring

In light of the above conceptual and ethical grounding, the WHO’s efforts to measure functional abilities of older people worldwide is both enormous and crucial for analytical, programmatic, and ethical reasons. We must be able to measure individual bodily (intrinsic) capacities, relevant external social conditions, and their interaction effects in order to accurately reflect the intra and inter-national levels and diversity in what individuals are actually able to be and do in their daily lives. There is a danger that we will continue to just measure intrinsic capacities ignoring surrounding conditions. Freedoms are also more difficult to measure than what individuals actually achieve. Subjective reports are also easier than observational studies. And many countries do not have the abilities to measure even what people achieve or say they achieve. But to begin to address the main policy problem of longevity without health, and the enormous injustices being done to older people—which the millions of pandemic deaths have visibly surfaced in recent years—we must strive to build robust and valid measurement and monitoring tools that capture health and wellbeing of older people, namely their ‘functional abilities’.

## References

[1] World Health Organization . World Report on Ageing and Health. Geneva: World Health Organization, 2015.

[2] Beard JR, Officer A, de Carvalho IA et al. The World report on ageing and health: a policy framework for healthy ageing. Lancet 2016; 387: 2145–54. 26520231 10.1016/S0140-6736(15)00516-4 PMC4848186

[3] Sen A . Capability and well-being. In: Nussbaum M, Sen A, eds. The Quality of Life. Oxford: Oxford University Press, 1993. 10.1093/0198287976.003.0003 (accessed 25 June 2023).

[4] The UN Decade of Healthy Ageing Secretariat. What is the UN Decade of Healthy Ageing (2021–2030)? https://www.decadeofhealthyageing.org/about/about-us/what-is-the-decade

[5] Rudnicka E, Napierala P, Podfigurna A et al. The World Health Organization (WHO) approach to healthy ageing. Maturitas 2020; 139: 6–11. 32747042 10.1016/j.maturitas.2020.05.018 PMC7250103

[6] The Economist . S. *Solstad (Corresponding Author); The Pandemic’s True Death Toll*. London: The Economist, 2021.

[7] Wong MK, Brooks DJ, Ikejezie J et al. COVID-19 mortality and progress toward vaccinating older adults — World Health Organization, worldwide, 2020–2022. MMWR Morb Mortal Wkly Rep 2023; 72: 113–8. 36730046 10.15585/mmwr.mm7205a1 PMC9927068

[8] Venkatapuram S . Health Justice : An Argument from the Capabilities Approach. Cambridge, UK; Malden, MA: Polity, 2011.

[9] Marmot M . Capabilities, human flourishing, and the health gap. In: Venkatapuram S, Broadbent A, eds. The Routledge Handbook of Philosophy of Public Health. London: Routledge, 2022. 10.4324/9781315675411-18.

[10] UK Parliamentary and Health Service Ombudsman. Introduction to the Social and Medical Models of Disability. London. https://www.ombudsman.org.uk/sites/default/files/FDN-218144_Introduction_to_the_Social_and_Medical_Models_of_Disability.pdf.

[11] Nussbaum MC . Capabilities and human rights. Fordham Law Rev 1997; 66: 273–300.

[12] British Geriatrics Society, Royal College of Physicians, Royal College of Physicians of Edinburgh et al. Protecting the rights of older people to health and social care. 2023. https://www.bgs.org.uk/policy-and-media/protecting-the-rights-of-older-people-to-health-and-social-care.

